# Antibacterial Activity of *Kalanchoe mortagei* and *K. fedtschenkoi* Against ESKAPE Pathogens

**DOI:** 10.3389/fphar.2019.00067

**Published:** 2019-02-06

**Authors:** Nicholas Richwagen, James T. Lyles, Brandon L. F. Dale, Cassandra L. Quave

**Affiliations:** ^1^Center for the Study of Human Health, Emory College of Arts and Sciences, Atlanta, GA, United States; ^2^Department of Dermatology, Emory University School of Medicine, Atlanta, GA, United States; ^3^Emory University Herbarium, Atlanta, GA, United States; ^4^Antibiotic Resistance Center, Emory University, Atlanta, GA, United States

**Keywords:** medicinal plants, MIC, phytochemicals, Crassulaceae, antibiotic resistance

## Abstract

Plants in the genus *Kalanchoe* (Family: Crassulaceae) are used in traditional medicine throughout the tropics for treating a variety of conditions. Two species, *Kalanchoe mortagei* and *K. fedtschenkoi*, have established ethnobotanical usage but have been neglected in previous research concerning their potential bioactivity. Here, we provide a thorough review of the reported antimicrobial activities of *Kalanchoe* genus and evaluate the *in vitro* antibacterial effects of two previously unexplored species against a panel of multidrug-resistant bacteria, the ESKAPE pathogens (*Enterococcus faecium, Staphylococcus aureus, Klebsiella pneumoniae, Acinetobacter baumannii, Pseudomonas aeruginosa*, and *Enterobacter cloacae*). Plant specimens were collected and voucher specimens deposited in the Emory University Herbarium. Dried plant material was ground into a powder and extracted as ethanolic macerations or as aqueous decoctions. Extracts were tested against the ESKAPE pathogens for growth inhibitory activity. Cytotoxicity to human cells was assessed via a lactate dehydrogenase assay of treated human keratinocytes (HaCaTs). *K. fedtschenkoi* extracts demonstrated growth inhibitory effects against two Gram-negative species, *A. baumannii* (strain CDC-33) and *P. aeruginosa* (AH-71), as well as *S. aureus* (UAMS-1). In these cases, growth inhibition greater than 50% (IC_50_) was generally observed at concentrations of 256 μg mL^-1^, though one *K. fedtschenkoi* extract (1465, prepared from stems) exhibited an IC_50_ against *A. baumannii* at 128 μg mL^-1^. All extracts were well tolerated by HaCaTs (LD_50_ ≥ 256 μg mL^-1^). Chemical characterization using HPLC and chemical standards established the presence of caffeic acid and quercetin in both plant species, as well as kaempferol in *K. fedtschenkoi.* These results reveal *K. fedtschenkoi* to be a plant of medicinal interest, and future research should aim to characterize the bioactivity of this species and its active constituents through bioassay-guide fractionation. Effects on bacterial biofilm formation and quorum-sensing are also research topics of interest for this genus.

## Introduction

### Ethnopharmacological Relevance of *Kalanchoe* Species

Plants in the genus *Kalanchoe* (Crassulaceae), though originating mostly in Madagascar and Southeast Africa, have a global distribution in warm climates. Frequently, *Kalanchoe* spp. occur as exotic or invasive species. Many members of the genus are able to self-propagate from plantlets produced on the leaf margin, making established populations hard to eradicate (Descoings, 2003; Akulova-Barlow, 2009). The presence of toxic cardiac glycosides make some *Kalanchoe* spp. a grazing hazard for animals in agriculture, with documented issues in Brazil, South Africa, and Australia (Botha C., 2013; Botha C.J., 2013; Mendonça et al., 2018). Nevertheless, these plants display a diverse array of stunning forms and are often grown as ornamentals for their strange beauty.

Despite their often exotic presence, *Kalanchoe* spp. have ethnobotanical uses wherever they are found, sometimes being called “miracle leaf” for their use in treating various ailments (Akulova-Barlow, 2009; Milad et al., 2014). In the developing world, members of this genus are used for treating myriad medical conditions. Because of its widespread distribution and ubiquitous ethnobotanical use, much research has been focused on *K. pinnata*, a species native to Madagascar but cultivated and distributed throughout the tropics (Descoings, 2003; Biswas et al., 2011a; Quazi Majaz et al., 2011; Pattewar, 2012; Rajsekhar et al., 2016). This species has even been the subject of bioengineering – a transgenic *K. pinnata* that produces an antimicrobial peptide (AMP cecropin P1) has recently been developed (Zakharchenko et al., 2016; Lebedeva et al., 2017).

Because the genus has demonstrated medicinal potential, *Kalanchoe* spp. neglected in research should be explored for bioactive compounds. *K. mortagei* and *K. fedtschenkoi*, two members of the section Bryophyllum within the genus, are two such species with established ethnobotanical usage, but which have been overlooked in natural products research.

*Kalanchoe mortagei*, also known by the synonyms *K. poincarei* or *Bryophyllum mortagei*, is a plant native to rocky/sandy soils in north Madagascar (Descoings, 2003). Compared to other members of the genus, little research has been conducted on the chemical and medicinal properties of this species (Maiti et al., 1995). Despite this, *K. mortagei* is grown in Mexican homegardens, and its leaves are taken orally for digestive disorders and as a local remedy for cancer in Antioquia Department, Colombia (Blanckaert et al., 2004; Vera-Marín and Sánchez-Sáen, 2016). The roots of the plant are used for treating parasitic worm-related diseases in parts of Indonesia (Herawati and Husin, 2000).

*Kalanchoe fedtschenkoi* is a perennial native to central/southern Madagascar but is naturalized well outside its original range (Descoings, 2003). Introduced populations can be found in Florida, Texas, and Puerto Rico (USDA/NRCS, 2013). A popular garden succulent, *K. fedtschenkoi* is a model organism for research into Crassulacean acid metabolism (CAM) (Dittrich, 1976; Nimmo et al., 1986; Cook et al., 1995). In Brazil, this species is used as an analgesic (Cumberbatch, 2011).

### Antimicrobial Resistance in the ESKAPE Pathogens

The rise of antimicrobial resistant (AMR) bacterial infections is one of the most pressing issues in medicine. Increasingly, conventional antibiotic medications are failing to stop persistent and dangerous bacterial diseases (Irenji et al., 2018; Katsuura et al., 2018). A report commissioned by the UK government notes that roughly 700,000 people die annually from AMR infections; this figure is projected to increase to 10 million deaths per year by 2050 (O’Neil, 2016) and encompasses data from across the broad spectrum of pathogenic microbes. In the face of rising morbidity and mortality due to AMR infections, the need for new drugs to address drug-resistance is clear (van der Meer et al., 2014). In 2015, the WHO launched the Global Antimicrobial Resistance Surveillance System (GLASS) to unify worldwide AMR. To date they have collected data from 42 countries and received over 500,000 AMR pathogenic strains (WHO, 2017).

Six bacterial species, the “ESKAPE” pathogens, have been highlighted by the Infectious Disease Society of America (IDSA) as being especially dangerous due to their patterns of antibiotic resistance. They are responsible for the majority of nosocomial infections worldwide (Table 1) (Boucher et al., 2009).

**Table 1:** Description of the ESKAPE pathogens.

	Species	Gram	Drug development needs (Boucher et al., 2009)
E	*Enterococcus faecium*	+	(VRE) Third most frequent cause of nosocomial blood borne infections. Increasing vancomycin resistance.
S	*Staphylococcus aureus*	+	(MRSA) Need for oral treatment agents, less cytotoxic drugs; current drugs subject to emerging resistance. Need for non-drug therapies.
K	*Klebsiella pneumoniae*	–	Can produce extended-spectrum beta-lactamases (ESBL) or are carbapenem-resistant; ESBL is associated with increased mortality and delay of effective therapy.
A	*Acinetobacter baumannii*	–	Rising global incidence of infection, can be carbapenem-resistance, increased mortality for burn patients. Serious absence of available treatment options.
P	*Pseudomonas aeruginosa*	–	Rising incidence; resistance to carbapenems, quinolones, polymyxins.
E	*Enterobacter* spp.	–	Rising incidence, ESBL, carbapenem-resistance.


### *Kalanchoe* Spp. as a Source of Antimicrobial Treatment

Plants used in traditional medicine are a potential source for novel antimicrobial compounds (Rahman et al., 2018; Salam and Quave, 2018). In the developing world, the large majority of people (75%) rely on plants for primary medical needs, including for wound healing and antimicrobial agents (Sarker et al., 2005). Historically, the bulk of manufactured drugs were derived from plant natural products, and the majority of these drugs were tied directly to their original ethnobotanical use (Chin et al., 2006; Sarker and Nahar, 2012). Even between 1982 and 2002, 79% of approved drugs worldwide had a natural product origin (Chin et al., 2006).

Secondary metabolites taken from plants used in traditional medicine have been found to inhibit microbial growth and virulence. *Kalanchoe* spp. have demonstrated such antimicrobial properties, and have been proven to accelerate wound-healing. For example, extracts and compounds from *K. pinnata* are effective against cutaneous leishmaniasis, a disease caused by trypanosome protozoa (Torres-Santos et al., 2003; Muzitano et al., 2006a,b, 2009).

In the past decade, substantial research has examined the antibacterial properties of *K. pinnata* and several other *Kalanchoe* spp. Extracts of *K. blossfeldiana, K. crenata, K. laciniata*, and *K. pinnata* have all demonstrated growth inhibitory effects on over 15 bacterial species, including four of the ESKAPE pathogens (Tables 2, 3).

**Table 2:** Literature review of research on the antimicrobial properties of *Kalanchoe* spp.

*Kalanchoe* sp.	Method	Microbes tested/Gram (+/-)	Results
*K. pinnata* (Kouitcheu Mabeku et al., 2017)	Leaf methanol and ethyl acetate extracts were tested against *Helicobacter pylori in vitro* and in the guts of Swiss mice.	*Helicobacter pylori* (-)	Methanol extract showed a significant anti-Helicobacter activity with MIC and MBC values of 32 and 256 μg mL^-1^, respectively. Also reduced bacterial load of gastric mucosa.
*K. pinnata* Transgenic and wild-type (Lebedeva et al., 2017)	Leaf aqueous extracts of wild-type and transgenic (cecropin producing) were applied directly to infected wounds.	Wounds were infected with *Staphylococcus aureus* (+), *Pseudomonas aeruginosa* (-), or a combination of both.	Both wild-type and transgenic extracts accelerated wound-healing and demonstrated anti-microbial effects, even in comparison to an antibiotic.
*K. pinnata* (Larasati and Wahid, 2016)	Leaf ethanolic extracts tested using microdilution method	*Acinetobacter baumannii* (-) and *S. aureus* (+)	Effective against both bacteria.
*K. laciniata* (Iqbal et al., 2016)	Aerial parts in a 60% methanolic extract	*S. aureus* (+) and *Bacillus subtilis* (+)	In assays the crude extract was found effective against *S. aureus* and *B. subtilis*, with MIC values of 5 and 2.5 mg mL^-1^, respectively.
*K. blossfeldiana* (Sarkar et al., 2015)	Methanolic extract evaluated against biofilm production	*P. aeruginosa* (-)	Extract reduced biofilm formation and thickness reduced secretion of virulence factors. Concentrated extract destroyed biofilms.
*K. pinnata* (Pattewar et al., 2013)	Leaf 95% ethanolic, methanolic extracts 60% methanolic, aqueous extracts	*S. aureus* (+), *P. aeruginosa* (-), *Escherichia coli* (-), and fungus *Candida albicans*	Zones of inhibition, MICs established (30 mg for *S. aureus*). All extracts showed antimicrobial effects. 60% methanol extracts performed best.
*K. pinnata* (Tatsimo et al., 2012)	Evaluation of methanolic, ethanolic crude extracts, and extract partitions (in ethyl acetate, hexane)	*S. aureus* (+), *P. aeruginosa* (-), *Salmonella typhi* (-) Fungi *C. albicans, Candida parapsilosis, Cryptococcus neoformans*	Crude extracts displayed strong antibacterial and especially antifungal effects. Ethyl acetate fractions more strongly anti-microbial. An isolated flavonoid showed particularly strong effects.
*K. pinnata* (Biswas et al., 2011b)	Ethanolic extracts used in agar-diffusion method.	*Bacillus megaterium* (+), *B. subtilis* (+), *S. aureus* (+), *E. coli* (-), *P. aeruginosa* (-), *Shigella dysenteriae* (-), *S. typhi* (-), *Vibrio cholera* (-)	Bacterial growth was inhibited by extract, expect for, *S. typhi, V. cholera*. Effects were strongest against *E. coli*, with a zone of inhibition of 8.2 ± 0.22.
*K. pinnata* (Majaz et al., 2011)	Root extracts of petroleum ether, chloroform, methanol, and water	*S. aureus* (-), *E. coli* (-), *P. aeruginosa* (-) Fungus *C. albicans*.	Methanolic extracts most effective against all bacteria; no extracts effective against *C. albicans*.
*K. pinnata* (Okwu and Nnamdi, 2011)	Two flavonoid compounds were isolated and tested directly	*P. aeruginosa* (-), *Klebsiella pneumoniae* (-), *E. coli* (-), *S. aureus* (-) Fungi *C. albicans and Aspergillus niger*	Zones of inhibition, MICs established for all bacteria tested.
*K. pinnata* (Nwadinigwe, 2011)	Stem extracts of methanol, water. Agar-diffusion	*S. typhi* (-), *P. aeruginosa* (-), *S. aureus* (+), *Bacillus subtilis* (+), Fungi *C. albicans and A. niger*	Bactericidal effects established against *B. subtilis and S. aureus*, with the methanolic extract showing strong effects. No effects *against P. aeruginosa, C. albicans*, and *A. niger*. *S. aureus* showed the lowest minimum inhibitory concentration (MIC) of 6.29 mg mL^-1^ in the methanol extract, while *S. typhi* showed the highest MIC of 9.98 mg mL^-1^ in the aqueous extract.
*K. crenata*/*K. pinnata* (Akinsulire et al., 2007)	Methanol, aqueous extracts. Juice from squeezed leaves. Three solvents based on local alcoholic beverages. Agar diffusion, broth dilution methods to determine MIC.	*E. coli* (-) ATCC 25922, *P. aeruginosa* (-), *K. pneumoniae* (-), *Shigella flexneri* (-), *Salmonella paratyphi* (-), *Citrobacter* spp. (-) *S. aureus* (+) ATCC 25213, *Enterococcus faecalis* (+), *B. subtilis* (+) Fungus *C. albicans*	Methanolic extracts of both species were effective against all tested, though Gram-positive bacteria were more susceptible. Aqueous extracts were less effective. *K pinnata* water extracts did not affect *E. coli, K. pneumoniae, S. paratyphi, Citrobacter*. Aqueous for either species did not affect *C. albicans*. Local solvents were not effective. Leaf juice extract was effective, particularly for *K. crenata*, against all except *C. albicans*.
*K. pinnata* (Ofokansi et al., 2005)	Methanolic extracts. Agar-diffusion, checkerboard.	*S. aureus* (+) ATCC 9637, *K. pneumonia* (-), *P. aeruginosa* (-), *S. typhi* (-), *E. coli* ATCC 9637	MIC determined against *S. aureus* and *B. subtilis, K. pinnata* demonstrated synergistic antibacterial effects with another plant
*K. pinnata* (Akinpelu, 2000)	60% methanolic extracts, tested at 25 mg mL^-1^	*S. aureus* (+), *K. pneumoniae* (-), *P. aeruginosa* (-), *E. coli* (-), *B. subtilis* (-), *S. dysenteriae* (-), *C. albicans*	*B. subtilis, E. coli, P. vulgaris, S. dysenteriae, S. aureus* were growth inhibited. *K. pneumoniae* and *P. aeruginosa* were not growth inhibited.
*K. pinnata* (Obaseiki-Ebor, 1985)	Leaf juice extract 5% v/v tested	*S. aureus* (+), *Streptococcus pyogenes* (+), *E. faecalis* (+), *E. coli* (-), *Proteus* spp. (-), *Klebsiella* spp. (+), *Shigella* spp. (-), *Salmonella* spp. (-), *Serratia marcescens* (-), *and P. aeruginosa* (-)	Bactericidal effects against all demonstrated.


*Almost all antimicrobial work has focused on the species *K. pinnata*. Table, in part, adapted from review papers: Biswas et al. (2011a), Quazi Majaz et al. (2011), Pattewar (2012), and Rajsekhar et al. (2016).*

**Table 3:** Review of *Kalanchoe* extracts tested against selected bacteria in previous research.

Bacteria/plant, paper, and solvent count	Methanolic extract	Water extract	Other
***Acinetobacter baumannii*** (-) *K. pinnata*: 1 paper, 1 extract solvent			***K. pinnata*** • Ethanol✓ (Larasati and Wahid, 2016)
*Bacillus subtilis* (+) *K. pinnata, K. laciniata*: 4 papers, 3 extract solvents	***K. pinnata*** ✓+ (Akinpelu, 2000; Nwadinigwe, 2011) ***K. laciniata*** ✓ (Iqbal et al., 2016)	***K. pinnata*** ✓ (Nwadinigwe, 2011)	***K. pinnata*** • Ethanol✓ (Biswas et al., 2011b)
***Enterobacter* spp.** (-) *K. pinnata*: 1 paper examining organic acid extract			***K. pinnata*** • Malic acid✓ extracted from plant using decoction method, successful against *E. aerogenes* (Jazul, 1995)
*Enterococcus faecalis* (+) *K. pinnata, K. crenata*: 2 papers, 2 solvents, and leaf juice.	***K. pinnata*** ✓+ (Akinsulire et al., 2007) *K. crenata* ✓+ (Akinsulire et al., 2007)	***K. pinnata*** ✓(Akinsulire et al., 2007) *K. crenata* ✓ (Akinsulire et al., 2007)	***K. pinnata*** • Leaf juice✓ (Obaseiki-Ebor, 1985; Akinsulire et al., 2007) *K. crenata* • Leaf juice✓ (Akinsulire et al., 2007)
***Enterococcus faecium*** (+)	No *Kalanchoe* extracts previously tested against this species		
*Escherichia coli* (-) *K. pinnata, K. crenata*: 9 papers, 5 extract solvents, leaf juice, and flavonoid compounds	***K. pinnata*** ✓+ (Akinpelu, 2000; Ofokansi et al., 2005; Akinsulire et al., 2007; Majaz et al., 2011; Nwadinigwe, 2011) ***K. crenata*** ✓+ (Akinsulire et al., 2007)	***K. pinnata*** ✓ (Akinsulire et al., 2007; Majaz et al., 2011; Nwadinigwe, 2011; Pattewar et al., 2013)χ(Akinsulire et al., 2007) ***K. crenata*** ✓(Akinsulire et al., 2007)	***K. pinnata*** • Ethanol✓ (Biswas et al., 2011b; Pattewar et al., 2013) • Petroleum ether, chloroform^∗^ (Majaz et al., 2011) • Flavonoid compounds✓ (Okwu and Nnamdi, 2011) • Leaf juice✓ (Obaseiki-Ebor, 1985; Akinsulire et al., 2007) ***K. crenata*** • Leaf juice✓ (Akinsulire et al., 2007)
*Helicobacter pylori* (-) *K. pinnata*: 1 paper, 2 solvents	***K. pinnata*** ✓+ (Kouitcheu Mabeku et al., 2017)		***K. pinnata*** • Ethyl acetateχ (Kouitcheu Mabeku et al., 2017)
***Klebsiella pneumoniae*** (-) *K. pinnata, K. crenata*: 5 papers, 2 solvents, leaf juice, and flavonoid compounds	***K. pinnata*** ✓+ (Ofokansi et al., 2005; Akinsulire et al., 2007) χ(Akinpelu, 2000) ***K. crenata*** ✓+ (Akinsulire et al., 2007)	***K. pinnata*** χ(Akinsulire et al., 2007) ***K. crenata*** ✓ (Akinsulire et al., 2007)	***K. pinnata*** • Flavonoid compounds✓ (Okwu and Nnamdi, 2011) • Leaf juice✓ (Obaseiki-Ebor, 1985; Akinsulire et al., 2007) ***K. crenata*** • Leaf juice✓ (Akinsulire et al., 2007)
***Pseudomonas aeruginosa*** (–) *K. pinnata, K. crenata, K. blossfeldiana*: 11 papers, 6 solvents, leaf juice, and flavonoid compounds	***K. pinnata*** ✓+ (Ofokansi et al., 2005; Akinsulire et al., 2007; Majaz et al., 2011; Pattewar et al., 2013) χ (Akinpelu, 2000; Nwadinigwe, 2011) ***K. crenata*** ✓+ (Akinsulire et al., 2007) ***K. blossfeldiana*** ✓ (Sarkar et al., 2015) inhibited biofilm production^∗∗^	***K. pinnata*** ✓(Akinsulire et al., 2007; Majaz et al., 2011; Nwadinigwe, 2011; Pattewar et al., 2013) ***K. crenata*** ✓ (Akinsulire et al., 2007)	***K. pinnata*** • Ethanol✓ (Biswas et al., 2011b; Tatsimo et al., 2012; Pattewar et al., 2013) • Ethyl acetate✓+, hexaneχ *fractions* (Tatsimo et al., 2012) • Petroleum ether, chloroform^∗^ (Majaz et al., 2011) • Flavonoid compounds (Okwu and Nnamdi, 2011) ✓ • Leaf juice✓ (Obaseiki-Ebor, 1985; Akinsulire et al., 2007) ***K. crenata*** • Leaf juice✓ (Akinsulire et al., 2007)
*Proteus* spp. (–) *K. pinnata*: 1 paper testing leaf juice			***K. pinnata*** • Leaf juice✓ (Obaseiki-Ebor, 1985)
*Salmonella typhi* (–) *K. pinnata:* 5 papers, 5 solvents, and leaf juice	***K. pinnata*** ✓+ (Ofokansi et al., 2005; Nwadinigwe, 2011; Tatsimo et al., 2012)	***K. pinnata*** χ (Nwadinigwe, 2011)	***K. pinnata*** • Ethanolic (Biswas et al., 2011b; Tatsimo et al., 2012) • Ethyl acetate✓+, hexaneχ *fractions* (Tatsimo et al., 2012) • Leaf juice (on *Salmonella* spp.) (Obaseiki-Ebor, 1985)
*Shigella dysenteriae* (–) *K. pinnata*: 3 papers, 2 solvents, and leaf juice.	***K. pinnata*** ✓(Akinpelu, 2000)		***K. pinnata*** • Ethanol✓ (Biswas et al., 2011b) • Leaf juice✓ (on *Shigella* spp.) (Obaseiki-Ebor, 1985)
***Staphylococcus aureus*** (+) *K. pinnata, K. crenata*: 14 papers, 7 solvents, leaf juice, and flavonoid compounds	***K. pinnata*** ✓+ (Akinpelu, 2000; Ofokansi et al., 2005; Majaz et al., 2011; Nwadinigwe, 2011; Tatsimo et al., 2012; Pattewar et al., 2013; Lebedeva et al., 2017) *K. crenata* ✓+ (Akinsulire et al., 2007) *K. laciniata* (Iqbal et al., 2016)	***K. pinnata*** ✓ (Akinsulire et al., 2007; Majaz et al., 2011; Nwadinigwe, 2011; Pattewar et al., 2013) *K. crenata* ✓ (Akinsulire et al., 2007)	***K. pinnata*** • Ethanol✓ (Biswas et al., 2011b; Tatsimo et al., 2012; Pattewar et al., 2013; Larasati and Wahid, 2016) • Ethyl acetate✓+, hexaneχ *fractions* (Tatsimo et al., 2012) • Petroleum ether, chloroform (Majaz et al., 2011) • Flavonoid compounds✓ (Okwu and Nnamdi, 2011) • Leaf juice✓ (Obaseiki-Ebor, 1985; Akinsulire et al., 2007) *K. crenata* • Leaf juice✓ (Akinsulire et al., 2007)
*Serratia marcescens* (–) *K. pinnata*: 1 paper testing leaf juice			***K. pinnata*** Leaf juice✓ (Obaseiki-Ebor, 1985)


*All listed were crude extracts unless otherwise noted (see Tatsimo et al., 2012). ESKAPE pathogens and *Kalanchoe* species are listed in bold. Methanol and water are the two most commonly studied extract solvents. ✓ indicates demonstrated growth inhibition, ✓+ indicates superior performance compared to other tested solvents within the same study. χ indicates no significant growth inhibition demonstrated. Next to species name, bacteria are noted as Gram positive (+) or negative (-).*

In 15 studies that evaluated the antimicrobial effects of *Kalanchoe* spp., 12 focused solely on *K. pinnata*, one on *K. laciniata* (Iqbal et al., 2016) and one on *K. blossfeldiana*, a common household ornamental (Sarkar et al., 2015). A 2007 study compared the growth-inhibitory properties of *K. crenata* favorably with *K. pinnata* (Akinsulire et al., 2007).

Ten studies examined methanolic extracts, the most common solvent tested. Ethanol and water (five studies each) were also frequently used solvents. Research has established that methanolic crude extracts of *K. pinnata* outperform aqueous extracts in their growth-inhibitory effects (Akinsulire et al., 2007; Majaz et al., 2011; Nwadinigwe, 2011; Pattewar et al., 2013); this is also true for *K. crenata* (Akinsulire et al., 2007).

Studies also established the antibacterial effects of flavonoids extracted from *K. pinnata* (Okwu and Nnamdi, 2011; Tatsimo et al., 2012), as well as its leaf juice (Obaseiki-Ebor, 1985; Akinsulire et al., 2007). At least one study demonstrated the effects of *K. pinnata in vivo*, looking at how aqueous extracts accelerate the healing of wounds infected with *Staphylococcus aureus* and/or *Pseudomonas aeruginosa* (Lebedeva et al., 2017).

Research has firmly established *K. pinnata* as a plant of medicinal interest, and the overall genus continues to show promise as a potential source of antimicrobial, antibacterial compounds.

The aim of this study was to evaluate the antimicrobial potential of two previously neglected species: *K. mortagei* and *K. fedtschenkoi* against a panel of clinically relevant ESKAPE pathogens.

## Materials and Methods

### Plant Collection and Identification

Two plant species were used in this experiment. *Kalanchoe mortagei* plants were grown from a specimen collected by the first author (NR) in Bradenton, FL, United States, in May 2008 (27.468591, -82.577127). A single *K. fedtschenkoi* plant was procured from the University of Georgia Plant Biology Greenhouse in Athens, GA, United States, in 2015. All plant material used in this experiment came from plants propagated from these two mother specimens. Plants were grown in NR’s personal collection and at the Emory University Greenhouse. Voucher specimens of each species were deposited at the Emory University Herbarium (GEO), and species identification confirmed by Dr. Tharanga Samarakoon at GEO (Accession nos.: 22702 and 22474 for *K. fedtschenkoi* and *K. mortagei*, respectively). Specimens were digitized and are available for viewing on the SERNEC portal (SERNEC, 2018).

Bulk plant materials were harvested, dried in a dehumidification chamber, and homogenized in a Waring blender into a fine powder. Retention vouchers of dried and ground material were prepared for future reference and stored in Quave Research Group laboratories at Emory University.

### Preparation of Extracts

A total of seven crude extracts were prepared, four from *K. mortagei* and three from *K. fedtschenkoi* (Table 4). Each extract represented a particular plant part or combination of parts, though extract creation was also guided by limitations in available plant biomass.

**Table 4:** Extracts of *K. mortagei* and *K. fedtschenkoi* used in this study.

Extract number	Species extracted	Plant part extracted	Extraction solvent	Yield (%)	Total phenolic content (mg GAE/g)
1420	*K. mortagei*	Leaves, stems (aerial parts), immature inflorescences	80% EtOH	19.62	331 ± 33
1468	*K. mortagei*	Leaves, stems (aerial parts)	95% EtOH	6.98	571 ± 87
1508	*K. mortagei*	Mature inflorescence, flowers	95% EtOH	16.13	818 ± 19
1509aq	*K. mortagei*	Mature inflorescence, flowers	H_2_O	22.25	1340 ± 116
1421	*K. fedtschenkoi*	Aerial parts (including woody stems)	80% EtOH	12.69	370 ± 17
1465	*K. fedtschenkoi*	Woody stems	95% EtOH	7.44	498 ± 50
1469	*K. fedtschenkoi*	Aerial parts (no woody stems)	95% EtOH	15.54	486 ± 6


*Comparing the fresh biomass of each plant to its dry mass showed that the non-water portion comprised 7.90 and 5.78% of the overall mass for *K. mortagei* and *K. fedtschenkoi*, respectively. The TPC is expressed in mg GAE/g dry extract.*

Dry, ground plant biomass was double macerated for 72 h each with either 80 or 95% ethanol at a 1:10 ratio (w/v). The extracts were agitated daily and then vacuum filtered. The aqueous extract (1509aq) was prepared as a decoction; the dry plant material was boiled with deionized water (dH_2_O) for 20 min and then filtered. After filtration the solvent was removed by rotary evaporation at ≤40°C. Extracts were redissolved in dH_2_O, shell frozen in a dry ice-acetone bath, and then lyophilized overnight on a Labconco FreeZone 2.5 Lyophilizer (Kansas City, MO, United States). Dry extracts were scraped into scintillation vials and stored at -20°C. Organic extracts were dissolved in DMSO and the aqueous extract was re-dissolved in dH_2_O to yield a stock concentration of 10 mg mL^-1^ for microbiological assays.

### Antibacterial Testing

#### Bacterial Strains and Cultures

Seven extracts (Table 4) were tested against strains of ESKAPE pathogens (Table 5). Two species were Gram-positive, *Enterococcus faecium* (EU-44) and *S. aureus* (UAMS-1); the rest were Gram-negative: *Klebsiella pneumoniae* (CDC-16), *Acinetobacter baumannii* (CDC-33), *P. aeruginosa* (AH-71), and *Enterobacter cloacae* (CDC-08). Strains were streaked from freezer stock onto tryptic soy agar (TSA) plates and incubated at 37°C overnight. Liquid cultures in tryptic soy broth (TSB) were made from individual plate colonies in 14 mL test tubes and were also incubated at 37°C overnight for use in growth inhibition assays.

**Table 5:** ESKAPE pathogens tested and their corresponding antibiotic resistance profiles as reported by the source provider (BEI Resources or CDC AR Bank) or as determined by antibiotic disc diffusion test (for AMC, IPM, PIP, RA, SXT, and TET) following CLSI breakpoints.

Species	Strain ID	Alternate ID	Antibiotic resistance profile^∗^	Other characteristics
*Enterococcus faecium*	EU-44	HM-959; Strain 513	AMC, RIF, SXT, TET, TZP	
*Staphylococcus aureus*	UAMS-1			Osteomyelitis isolate; MSSA; prototype biofilm isolate
*Klebsiella pneumoniae*	CDC-16	AR-Bank #0016	AMP, ATM^I^, FOX, SAM^I^, TET	Reduced susceptibility, elevated carbapenem MICs
*Acinetobacter baumannii*	CDC-33	AR-Bank #0033	CAZ, CIP, CRO, CTX, DOR, FEP, GEN, IPM, LVX, MEM, SAM, SXT, TOB, TZP	Reduced susceptibility, elevated carbapenem MICs
*Pseudomonas aeruginosa*	AH-0071	PAO1		
*Enterobacter cloacae*	CDC-08	AR-Bank #0008	AMC, AMP, ATM, CAZ, CFZ, CIP, CRO, CTX, DOR^I^, ETP, FOX, LVX, MEM^I^, SAM, TET, TZP	Reduced susceptibility, elevated carbapenem MICs


*^∗^Resistance: AMC, amoxicillin–clavulanic acid; AMP, ampicillin; ATM, aztreonam; CAZ, ceftazidime; CIP, ciprofloxacin; CRO, ceftriaxone; CTX, cefotaxime; DOR, doripenem; ETP, ertapenem; FEP, cefepime; FOX, cefoxitin; GEN, gentamicin; IPM, imipenem; LVX, levofloxacin; MEM, meropenem; RIF, rifampicin; SAM, ampicillin-sublactam; SXT, trimethoprim–sulfamethoxazole; TET, tetracycline; TOB, tobramycin; TZP, piperacillin–tazobactam. Any antibiotics denoted with ^I^ indicates resistance.*

#### Growth Inhibition Assays

The extracts were examined for the growth inhibitory activity following guidelines set by the Clinical and Laboratory Standards Institute for broth microdilution testing (CLSI, 2013). After incubation, TSB cultures were diluted in cation-adjusted Muller Hinton broth (CAMHB) based on their optical density (OD_590_) to a confluence of 5 × 10^5^ CFU mL^-1^, confirmed by plate counts. All assays were performed in CELLSTAR 96-well plates (Greiner Bio-One International, 655-185), and read in a Cytation-3 multimode plate reader (BioTek). An initial optical density reading was taken after bacterial cultures and extracts were added to each plate (OD_600_). For *E. faecium, S. aureus, K. pneumoniae, P. aeruginosa*, and *E. cloacae*, assay plates were incubated for 18 h; *A. baumannii* was incubated for 22 h. After incubation, the optical density of wells was checked again (OD_600_).

In the initial screen, each extract was tested at a concentration of 256 μg mL^-1^ to determine if any growth-inhibitory effects at a level of 50% or greater were evident in comparison to the vehicle (DMSO) control. If bacterial growth was inhibited by at least 50%, microdilution assays were performed. Dose response studies were performed on bacteria-extract pairs exhibiting ≥50% growth inhibition in this initial screen. Extracts were tested by twofold serial dilution at a concentration range of 8–256 μg mL^-1^.

Percent inhibition was calculated in order to minimize the influence of any color cast due to the plant extracts as previously described (Quave et al., 2008). The IC_50_ values were defined as the concentration required to achieve a 50% inhibition of growth, and the MIC values (or IC_90_) were defined as the concentration required to achieve 90% growth inhibition (as determined by OD_600_ for both values). Gentamicin was used as a positive control against all strains.

### Mammalian Cytotoxicity Assay

Mammalian cytotoxicity of extracts was assessed using human keratinocytes (HaCaTs) and a lactate dehydrogenase (LDH) test kit (G-Biosciences, St. Louis, MO, United States) as previously described (Quave et al., 2015). Briefly, HaCaTs were maintained in Dulbecco’s modified Eagle’s medium with L-glutamine and glucose supplemented with 10% heat-inactivated fetal bovine serum and 1× solution of penicillin and streptomycin at 37°C, 5% CO_2_ in 75 mL flasks. Once 90–95% confluency was reached, the cells were detached from the flask bottom using 0.25% trypsin, 0.1% ethylenediaminetetraacetic acid (EDTA) in Hanks’ balanced salt solution (HBSS) without Ca^++^, Mg^++^, and NaHCO_3_. The culture was standardized to 4 × 10^4^ cells mL^-1^ using a hemocytometer. Then, 200 μL of the standardized culture was added to each well in a 96-well tissue culture-treated microtiter plate (Falcon 35–3075) and the plates were incubated for 48 h in a humidified 37°C, 5% CO_2_ incubator, prior to media aspiration. Either media containing extracts (4–512 μg mL^-1^) or vehicle were serially diluted and processed 24 h later following manufacturer’s protocol for chemical induced cytotoxicity. Percent DMSO (v/v) in the wells was <2% for all tests.

### Chemical Characterization

Each extract was characterized by HPLC using a method adapted from four previously published HPLC methods, one examining flavonoid compounds (Nielsen et al., 2005), and three examining bufadienolides (a type of cardiac glycoside commonly found in *Kalanchoe* plants) (Supratman et al., 2000; Huang et al., 2013; Moniuszko-Szajwaj et al., 2016). Extracts were dissolved in methanol (1465, 1469), methanol:dH_2_O (1420, 1421, 1509aq), or methanol:dH_2_O:DMSO (1468, 1508). All extracts were chromatographed on an Agilent 1260 Infinity system running OpenLab CDS ChemStation (Agilent Technologies, Santa Clara, CA, United States) with an Agilent ZORBAX Eclipse XDB-C18 (250 mm × 4.6 mm, 5 μm) column with compatible guard column at 30°C. A 10 μL injection of each extract was eluted at a flow rate of 1 mL min^-1^ using a mobile phase consisting of (A) 0.1% formic acid in H_2_O and (B) 0.1% formic acid in methanol (VWR HiPerSolv CHROMANORM). The gradient profile consisted of initial conditions 98:2 A:B which were held for 20 min, then increased to 24.5:75.5 A:B from 20 to 95.5 min, and finally to 100% B at 110 min, which was held for 20 min. Chromatograms of each extract were generated using ultraviolet–visual spectroscopy (UV–vis) during HPLC, and reported at 254 nm.

Standard flavonoids, kaempferol (MP Biomedicals, Inc.), and quercetin (Enzo Life Sciences), as well as phenolic compounds, caffeic acid, *p*-coumaric acid, and ferulic acid (MP Biomedicals, Inc.) were used to aid in characterization by HPLC.

### Detection of Total Phenolic Content

Total phenolic content (TPC) was determined using a Folin–Ciocalteu assay modified for 96-well plate format (Singleton et al., 1999). A 1 mg mL^-1^ gallic acid stock solution was prepared in 50% MeOH_(aq)_ and diluted in the same solution to yield 0–100 μg mL^-1^ gallic acid standard solutions. Extracts were prepared at 1 or 2 mg mL^-1^ in 50% MeOH_(aq)_ and serially diluted until their absorbance was within the range of the gallic acid standard curve. In a 96-well plate, 30 μL of gallic acid standard solution or extract was added to triplicate wells. To each well 200 μL of dH2O was added, then 15 μL of Folin–Ciocalteu regent. After at least 1 min, but no more than 8 min, 50 μL of 20% Na_2_CO_3_ (w/v) was added to all wells. The plate was mixed for 30 s on an orbital shaker, incubated at 40°C for 30 min, manually mixed with a multichannel pipette, then an additional 30 s with an orbital shaker, and finally the absorbance at 760 nm was recorded using a BioTek Cytation 3 multimode plate reader. The linear range for the assay was determined as 0–100 μg mL^-1^ gallic acid equivalents (GAE), *R*^2^ = 0.986. The TPC of the extracts is expressed as mg GAE/g dry extract.

## Results

### *K. fedtschenkoi* Exhibits Antibacterial Activity Against Three ESKAPE Pathogens

Initial screening of extracts at 256 μg mL^-1^ demonstrated an IC_50_ (growth inhibition of 50% or greater) of *K. fedtschenkoi* extracts (1421, 1465, and 1469) against three of the ESKAPE pathogens: *S. aureus, A. baumannii*, and *P. aeruginosa*. Further testing by serial dilution assays revealed that *K. fedtschenkoi* extracts had IC_50_ values ranging from 128 to 256 μg mL^-1^ for these pathogens (Table 6).

**Table 6:** Extracts exhibiting IC_50_ growth inhibition (≥50%) against ESKAPE pathogens.

		*E. faecium*	*S. aureus*	*K. pneumoniae*	*A. baumannii*	*P. aeruginosa*	*E. cloacae*
		
Species	Extract ID	EU-44	UAMS-1	CDC-16	CDC-33	AH-71	CDC-08
*K. mortagei*	1420	>256	>256	>256	>256	>256	>256
	1468	>256	>256	>256	>256	>256	>256
	1508	>256	>256	>256	>256	>256	>256
	1509aq	>256	>256	>256	>256	>256	>256
*K. fedtschenkoi*	1421	>256	>256	>256	**256**	>256	>256
	1465	>256	**256**	>256	**128**	**128**	>256
	1469	>256	**256**	>256	**256**	**256**	>256
Gentamicin MIC		8	16	>64	>64	4	<4


*Growth inhibition is in comparison to vehicle control. All concentration values reported as μg mL^-*1*^. Extracts active at an IC_*50*_ of 256 μg mL^-*1*^ or less are displayed in bold.*

**FIGURE 1:**
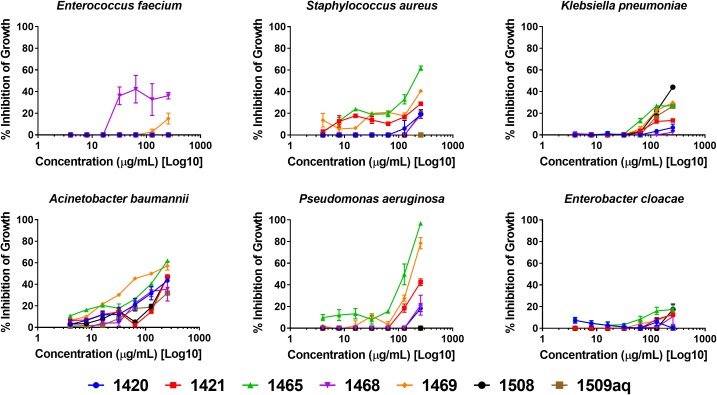
Growth inhibitory activity of *Kalanchoe* spp. extracts.

Growth inhibition by dose response is reported in Figure 1. The only extract to exhibit >35% inhibition in *E. faecium* (EU-44) was 1468. No extracts inhibited growth of *E. cloacae* (CDC-08) by 20% or more. Extract 1465 (*K. fedtschenkoi* woody stems) exhibited >60% inhibition in *A. baumannii* (CDC-33) and an MIC of 256 μg mL^-1^ (growth inhibition ≥ 90%) was observed against *P. aeruginosa* (AH-71). Extract 1508 was the only extract to exhibit at least 40% inhibition in growth at 256 μg mL^-1^ against *K. pneumoniae.*

### Extracts Exhibit Low Toxicity to Human Keratinocytes

**FIGURE 2:**
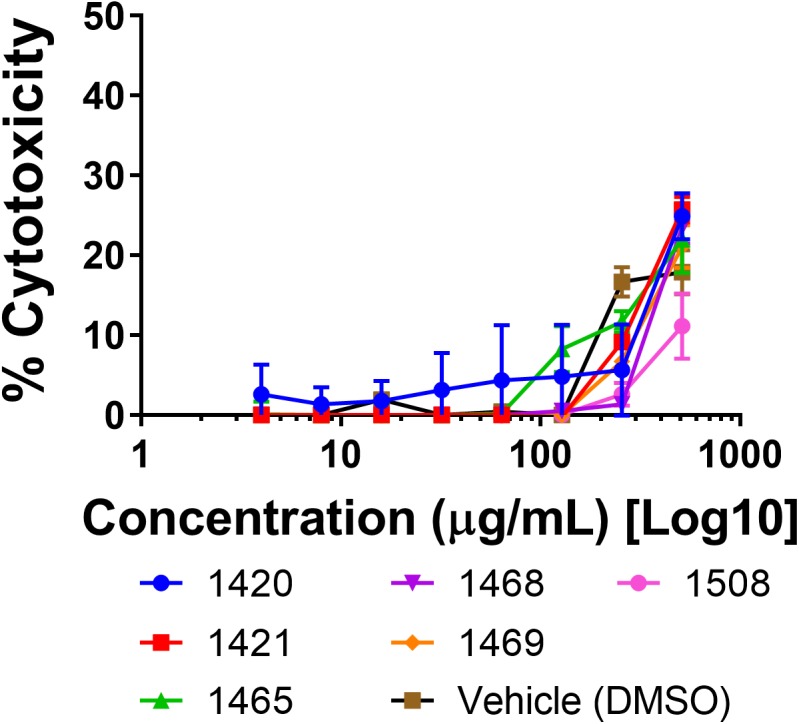
Cytotoxicity of extracts in a human keratinocyte (HaCaT) cell line by LDH assay for cell viability.

Human skin keratinocytes (HaCaTs) were exposed to each extract to examine possible cytotoxic effects in mammalian cells. The highest levels of cytotoxicity were observed at the 512 μg mL^-1^ concentration, and ranged from 11 to 26% growth inhibition of human cells. All extracts at the 256 μg mL^-1^ concentration exhibited cytotoxicity of 12% or less (Figure 2). No IC_50_ was observed for any of the tested concentrations.

### Chemical Characterization of Extracts

**FIGURE 3:**
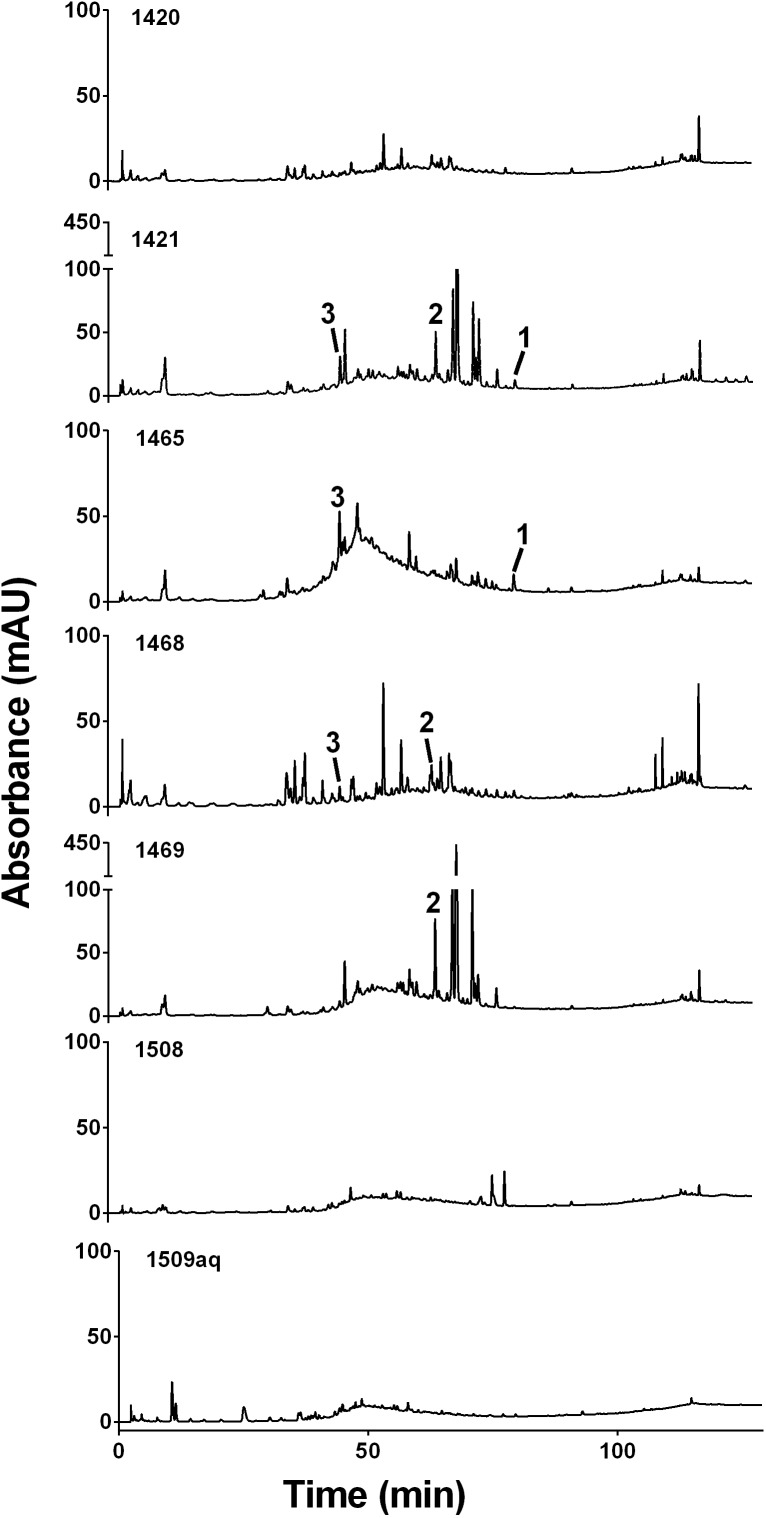
HPLC chromatograms at 254 nm for each *Kalanchoe* extract indicating compounds identified: kaempferol (**1**), quercetin (**2**), and caffeic acid (**3**).

In this study, the *Kalanchoe* spp. extracts were screened by HPLC for the presence of several commonly occurring flavonoids; kaempferol (**1**) and quercetin (**2**), and phenolic compounds, caffeic acid (**3**), *p*-coumaric acid (**4**), ferulic acid (**5**). Both *K. mortagei* (extracts 1421 and 1469) and *K. fedtschenkoi* (extract 1468) contained **2**. The extracts 1421 and 1465 of *K. fedtschenkoi* contained **1**. The presence of **3** was also established in *K. fedtschenkoi* (extracts 1421 and 1465) and in *K. mortagei* (extract 1468) at very low levels (Figure 3).

Previous studies have shown a diverse chemistry in the genus *Kalanchoe*. Previous studies identified **5** in several *Kalanchoe* species (Gaind and Gupta, 1971; Muzitano et al., 2006a,b; Cruz et al., 2012). **1** was established in *K. pinnata* (Gaind and Gupta, 1971; Muzitano et al., 2006a) and *K. daigremontiana* (Ürményi et al., 2016). Syringic acid, **3**, and **4** were identified in *K. pinnata* (Gaind and Gupta, 1973). A 1995 study found lupeol, lupeol acetate, β-sitosterol, and other related compounds in *K. mortagei* (Maiti et al., 1995).

The TFC for the *Kalanchoe* spp. extracts ranged from a minimum of 331 ± 33 mg GAE/g extract for 1420 to 1340 ± 116 mg GAE/g extract for 1509aq. The *K. mortagei* inflorescences extracts (1508 and 1509aq) had higher TFC than the other plant parts of both species, 818 mg GAE/g extract and 1340 mg GAE/g extract, respectively. The average TFC of the *K. mortagei* extracts with leaf and stem tissues (1420 and 1468) and the *K. fedtschenkoi* leaf and stem tissue extracts (1420 and 1465) were both 451 mg GAE/g extract, indicating that both species have similar TFC. However, the *K. fedtschenkoi* leaf and stem tissue extracts (1421, 1465, and 1469) have higher antimicrobial activities against multiple bacterial strains than the *K. mortagei* extracts. This suggests that the bioactivity of *K. fedtschenkoi* is not due to phenolic compounds.

## Discussion

In this study, *K. fedtschenkoi* extracts exhibited growth inhibition against two Gram-negative species, *A. baumannii* (CDC-33) and *P. aeruginosa* (AH-71), as well as Gram-positive *S. aureus*. All other pathogens examined, including Gram-positive *E. faecium* (EU-44), were largely unaffected. This contrasts with some previous work, where *Kalanchoe* spp. extracts tested against bacteria exhibited growth-inhibitory effects more readily against Gram-positive pathogens (Akinsulire et al., 2007). Extracts in other studies with *S. aureus* have always shown growth-inhibition, with the exception of the poor-performance of a hexane fraction tested (Tatsimo et al., 2012; Table 3). Tests against Gram-negative species *P. aeruginosa* and *K. pneumoniae* have had mixed results, demonstrating both positive (Akinsulire et al., 2007; Pattewar et al., 2013) and negative (Akinpelu, 2000; Nwadinigwe, 2011) results concerning growth-inhibition.

Although *K. mortagei* extracts 1420 and 1468 failed to inhibit growth at or above 50% (IC_50_), there were differences in performance and chemical characterization of these two closely related extracts. Both 1420 and 1468 were composed of aerial parts of *K. mortagei* (leaves and stems), though 1420 also had immature inflorescences. Against *E. faecium* (EU-44) and *P. aeruginosa* (AH-71), 1420 actually increased bacterial growth, and against all six pathogens, there were statistically different performances between these two extracts (verified with Student’s *t*-test). HPLC analysis revealed lower absorbance intensity in the 35–80 min region for 1420 compared to 1468, though elution peaks were similar. Caffeic acid could only be confirmed in 1420, and kaempferol was only confirmed in 1468.

It is possible that the differences are due to the harvest conditions of the *K. mortagei* plants used to make these extracts. Extract 1420 was prepared from a *K. mortagei* plant collected in December 2017, which was maintained in low-light conditions. Extract 1468, in contrast, was collected in March 2018 and was grown in bright light in a greenhouse setting. Research has shown that the chemical composition of *K. pinnata* is dependent on the plant’s light, growth, and harvest conditions; in bright light, the concentration of quercetin increased sevenfold, and that flavonoid compounds were more abundant during summer months (Muzitano et al., 2011). It is possible that the suboptimal growth conditions of the *K. mortagei* plant used for extract 1420 prevented the production of certain bioactive secondary metabolites.

## Conclusion

*Kalanchoe* is an important genus with relevance to traditional medicine across the globe. We have provided a comprehensive review of the reported antibacterial activities of *Kalanchoe* species, in particular *K. pinnata, K. crenata, K. blossfeldiana*, and *K. laciniata*. For the first time, we have reported the antibacterial activities of two understudied species in this genus (*K. fedtschenkoi* and *K. mortagei*) against clinically relevant, multidrug-resistant (MDR) strains of Gram-positive and Gram-negative bacteria. Our counterscreens against HaCaTs demonstrated that these extracts exhibit low toxicity to mammalian cells, supporting specificity of the action of these extracts against bacterial pathogens. Extracts were also characterized by HPLC, using chemical standards for peak identification and differentiation in their composition.

We demonstrated the antibacterial potential of *K. fedtschenkoi* against three ESKAPE pathogens. Particularly noteworthy was the specific growth-inhibition observed for *A. baumannii*, a Gram-negative species with rising global incidence that currently lacks sufficient treatment options (Boucher et al., 2009). In order to fully examine the potential of *K. fedtschenkoi* secondary metabolites, future work should aim to characterize the bioactivity of different extracts through bioassay-guided fractionation and isolation of active fractions and/or individual compounds. Additional studies should also look to address potential biofilm-inhibitory properties and interference in bacterial quorum sensing. *K. blossfeldiana* extracts have been shown to reduce biofilm growth or destroy biofilms entirely (Sarkar et al., 2015), and biofilm inhibition remains one of the most likely avenues for successful implementation of an anti-bacterial agent derived from plants (Wright, 2017). Other members of the genus *Kalanchoe* neglected in research should also be assessed for anti-microbial potential.

## Author Contributions

NR grew and collected the plant specimens, prepared the extracts, and performed the antibacterial experiments. JL and NR performed the chemical analysis of the extracts. BD performed the HaCaT cytotoxicity experiments. CQ designed and directed the study. NR and CQ analyzed the data and wrote the manuscript. All authors read, revised, and approved the final manuscript.

## Conflict of Interest Statement

The authors declare that the research was conducted in the absence of any commercial or financial relationships that could be construed as a potential conflict of interest.
